# Associations between 24-hour movement behavior and non-suicidal self-injury in adolescents: based on compositional data and isotemporal substitution analysis

**DOI:** 10.1186/s40359-026-05048-6

**Published:** 2026-07-06

**Authors:** Yingzhe Gao, Xiaoqiang Zhang, Huijuan Hu, Lunxin Chen, Hui Zeng

**Affiliations:** 1 School of Physical Education and Sports, Central China Normal University, No. 382 Xiongchu Avenue, Wuhan, 430079 China; 2 School of Physical Education, Wuhan Sports University, Wuhan, 430079 China; 3 School of Physical Education, Anhui Normal University, Wuhu, 241002 China

**Keywords:** Non-suicidal self-injury, Physical activity, Sedentary behavior, Sleep, Sompositional data analysis, Adolescents

## Abstract

**Background:**

Evidence suggests that physical activity (PA) is closely associated with lower non-suicidal self-injury (NSSI). However, the relationship between 24-h movement behaviors and NSSI, as well as potential substitution effects among these behaviors, remains unclear. Therefore, this study investigates how relative time allocation across movement behaviors and the substitution effects between them relate to NSSI.

**Methods:**

A total of 536 adolescents wore the ActiGraph wGT3X-BT triaxial accelerometer for 7 consecutive days to measure moderate-to-vigorous physical activity (MVPA), light-intensity physical activity (LPA), and sedentary behavior (SB); sleep duration was assessed via sleep diaries. NSSI scores were measured using the Self-Injury Behavior Scale (SIBS). Data were analyzed using compositional data analysis and the isotemporal substitution model.

**Results:**

Compositional regression showed a negative association between MVPA and NSSI scores and a positive association with SB. Isotemporal substitution using 15-minute increments indicated that replacing LPA and SB with MVPA was associated with reductions in NSSI scores of 0.31 and 0.37 units. Replacing SB with sleep was associated with a decrease in NSSI scores of 0.18 units. A significant interaction between sleep proportion and gender was observed, indicating sex-specific associations. Dose-response analysis further showed that substituting SB with MVPA produced the greatest reduction in NSSI, with effects attenuating after 20 min.

**Conclusion:**

Within the 24-h movement behavior framework, reallocating some SB time to MVPA may help reduce NSSI among adolescents. This finding improves our understanding of activity-related mechanisms in NSSI and informs targeted interventions.

**Supplementary Information:**

The online version contains supplementary material available at https://doi.org/10.1186/s40359-026-05048-6.

## Introduction

Non-suicidal self-injury (NSSI) refers to the deliberate destruction of one’s own body tissue without suicidal intent, such as cutting, stabbing, biting, or burning [1]. Adolescence is a critical period for the onset and escalation of NSSI, with peak incidence typically occurring between 12 and 15 years [2]. Meta-analytic evidence estimates a global prevalence of 17.2% among adolescents [3], with higher rates reported in Chinese samples (27.2%) [4]. NSSI is strongly associated with multiple adverse mental health outcomes and is a robust predictor of suicidal ideation and suicide attempts [5, 6]. Given its prevalence and clinical consequences, identifying modifiable behavioral risk factors is essential for prevention.

From the perspective of emotion regulation theory, NSSI is conceptualized as a maladaptive strategy used to downregulate intense negative affect when adaptive regulatory resources are insufficient [1, 7]. It reflects deficits in emotion regulation capacity, whereby self-injury provides short-term relief from aversive emotional states [8, 9]. Within this framework, daily behavioral patterns may shape emotion regulation processes and thus influence NSSI risk. Physical activity (PA), sedentary behavior (SB), and sleep are key components of adolescents’ daily time use and are closely linked to emotional functioning [10, 11]. PA is associated with enhanced affect regulation, resilience, and stress coping, whereas SB is linked to rumination and psychological distress. Sleep insufficiency is consistently associated with heightened emotional reactivity and impaired self-regulation.

Empirical studies consistently indicate that PA is inversely associated with NSSI [12–14], with moderate-to-vigorous physical activity (MVPA) showing potential protective effects [15]. In contrast, SB is positively associated with NSSI in large adolescent samples [16], while insufficient sleep is also linked to increased self-injurious behaviors [17, 18]. However, most studies have examined these behaviors separately, ignoring their co-dependent structure within a fixed 24-hour period. Because time allocation is inherently constrained, PA, SB, and sleep should be considered as a compositional system in which relative distribution, rather than absolute duration, determines health outcomes.

This limitation reflects a broader mismatch between traditional statistical models and the structure of daily time use. Standard regression approaches assume independence among predictors and fail to account for relative and substitutive relationships among 24-hour movement behaviors [19]. Compositional data analysis (CoDA) provides a suitable framework for such constrained data by focusing on relative information within a closed composition [20]. In addition, isotemporal substitution models estimate the effects of reallocating time between behaviors under a fixed total time constraint, capturing meaningful behavioral trade-offs [21]. Although these methods have been increasingly applied in physical and mental health research [22–25], their application to adolescent NSSI remains limited. Given the high prevalence and clinical relevance of NSSI in adolescence, examining how 24-hour movement behavior compositions relate to NSSI is essential for prevention-focused research. A compositional framework is therefore necessary to better capture system-level associations between daily behaviors and NSSI.

Accordingly, this study used accelerometer-based data to assess adolescents’ 24-hour movement behavior composition and applied CoDA combined with isotemporal substitution modeling to examine associations with NSSI and the effects of time reallocation. This approach aims to clarify how integrated behavioral patterns relate to NSSI and to inform intervention strategies. Grounded in emotion regulation theory and a 24-hour behavioral framework, we hypothesized that: (1) a healthier composition characterized by higher relative MVPA and lower relative SB would be associated with lower NSSI; (2) reallocating time from SB to MVPA would show the strongest beneficial association with NSSI.

## Methods

### Study sample

**Fig. 1:**
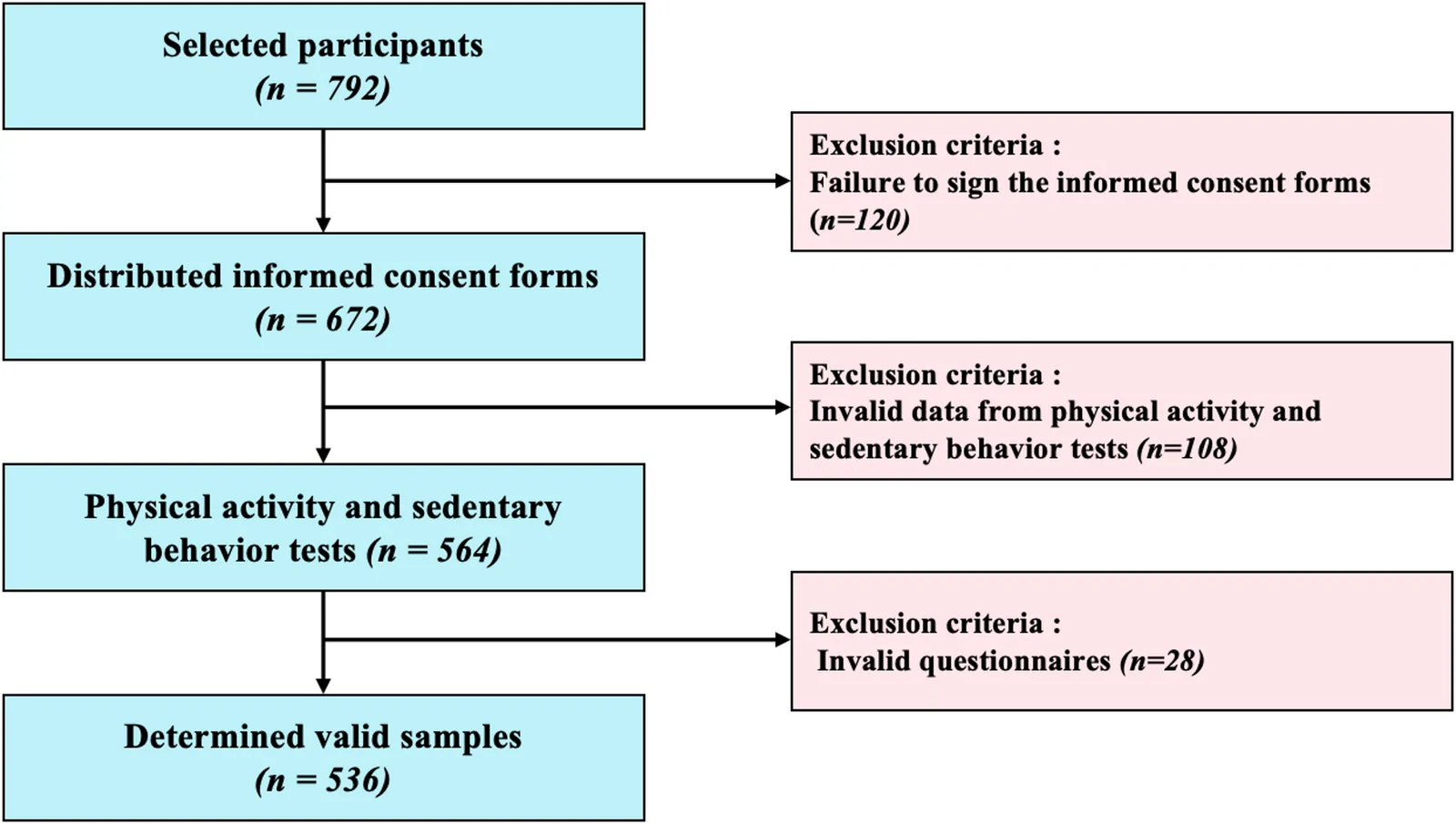
Flowchart of participants’ selection

From January to March 2025, a cross‑sectional survey was conducted in Wuhan, central China, using a stratified cluster random sampling approach. First, all junior high schools in Wuhan were divided into two strata based on geographic location. Two schools were randomly selected from each stratum, resulting in a total of four participating schools. Next, two classes were randomly selected from each of the 7th and 8th grades in each participating school, and all students in these classes were invited to participate. Eligible participants had no reading difficulties, no history of alcohol or substance abuse in the past six months, and no severe psychological or physical disorders. Written informed consent was obtained from both the students and their guardians. A 24‑h movement behavior assessment was conducted among 672 students who provided assent and whose parents provided written consent. After data collection, 108 participants were excluded due to incomplete or invalid accelerometer data, leaving 564 valid samples (validity rate: 83.93%). We then distributed questionnaires to these 564 students, which covered demographic information and the NSSI scale to assess self-injury behaviors. Finally, 536 valid questionnaires were collected, with an effective response rate of 95.03%. The detailed study flow is presented in Fig. 1.

### Measures

#### 24-hour movement behavior

Consistent with previous studies [26–28], this study classified 24-hour movement behaviors into four types, including sleep, SB, LPA, and MVPA. The total duration of these four behaviors reaches 1440 min every day. MVPA, LPA, and SB data during 24-hour movement were collected using the ActiGraph wGT3X-BT triaxial accelerometer (Actigraph LLC, Pensacola, FL, USA), while sleep was assessed via a daily sleep log completed by participants. Before data collection, researchers briefed participants on the purpose of the assessment, device-wearing protocols, and relevant precautions.

During the 7-day monitoring period (encompassing 5 school days and 2 weekend days), participants were instructed to wear the accelerometer on the right hip at all times, except during sleep and water-based activities (e.g., bathing, swimming). The raw accelerometer data were collected at a sampling frequency of 10 Hz. The research team conducted 2–3 in-person follow-ups during monitoring to ensure consistent and proper device wear. Following data collection, ActiLife 6.13.4 software was used for data cleaning and processing. Only data from participants with at least 3 valid monitoring days (2 weekdays and 1 weekend day) and a daily wear time of ≥ 10 h were included in subsequent analyses [29]. Non-wear time was identified using the standard algorithm proposed by Choi et al. [30]. Specifically, periods with zero accelerometer counts lasting no less than 60 min were defined as non-wear time, allowing brief fluctuations of 1 to 2 min with counts below 100 within such intervals. In this study, the average valid daily wear time during waking hours among participants was 12.34 ± 1.25 h.

PA intensity was categorized using ActiGraph count thresholds validated for adolescent populations [31]: 0-100 counts/min for SB, 101–2295 counts/min for LPA, 2296–4011 counts/min for moderate PA, and ≥ 4012 counts/min for vigorous PA. MVPA was defined as the combined duration of moderate and vigorous PA. This cut-point set has been validated among adolescents with favorable classification accuracy and applicability [32].

To minimize the potential impact of hip-worn accelerometers on students’ nocturnal sleep and to improve the accuracy of sleep duration estimation, sleep time in this study was determined by integrating sleep diaries with accelerometer-derived non-wear periods. Accelerometer data processed via ActiLife software were used to identify non-wear periods as an objective reference for sleep interval detection. The specific procedure was as follows: (1) participants completed a standardized sleep diary each day, reporting bedtime of the previous night and wake-up time of the current morning; (2) accelerometer data were processed to identify nocturnal non-wear periods, which served as an objective indicator for defining sleep intervals; (3) when self-reported sleep times closely matched the accelerometer-derived non-wear periods, sleep diary records were used as the primary source; (4) when discrepancies occurred between the two data sources, accelerometer-derived non-wear periods were adopted as the final estimate of sleep duration; and (5) nap duration was recorded in the sleep diary and incorporated into the total 24-hour activity time budget to provide a comprehensive representation of daily movement behaviors. This combined approach has been widely used in 24-hour movement behavior research and is suitable for large-scale epidemiological studies due to its balance between feasibility and data completeness [28, 33–37].

#### Non-suicidal self-injury

In the present study, NSSI was assessed using the Self‑Injury Behavior Scale (SIBS), a measure validated for use with Chinese adolescents [38]. The scale consists of seven items that evaluate various forms of NSSI, including cutting, burning, biting, and hitting oneself. Participants were asked: “In the past six months, have you engaged in the following behaviors to intentionally harm yourself without suicidal intent?” All seven items were rated on a 4‑point Likert scale (1 = never; 4 = six times or more). The total score of the scale was calculated, with higher scores indicating more severe self-injurious behavior. The scale has demonstrated good psychometric properties in previous research [39]. In the present study, the Cronbach’s α coefficient was 0.899.

#### Covariates

Based on previous research indicating that several sociodemographic and family-related factors are closely associated with NSSI in adolescents [40], this study included age, gender (boy, girl), parental educational level (middle school or below, high school or vocational school, college or above; when parental educational levels differed, the higher level was used), family economic status (low income, high income), place of origin (urban, rural), and body mass index (BMI) as covariates. Height and weight were measured using standardized international anthropometric procedures. A stadiometer (Seca) and a digital weight scale (Wujin, RGT-120) were used to measure height and weight, respectively. Participants were barefoot and wore light clothing to minimize measurement error. Both height and weight were measured twice, and the mean of the two readings was used as the final value. Body mass index (BMI) was calculated as weight (kg) divided by height squared (m²).

### Statistical analysis

Statistical analyses were conducted using RStudio version 4.4.2. Compositional data analysis and isometric log-ratio transformation were performed using the “compositions” and “robCompositions” packages in R [20].

1. Descriptive analyses. Descriptive statistics were used to summarize participants’ sociodemographic characteristics and the compositional variables of 24-hour movement behaviors. Both the arithmetic mean and the compositional geometric mean of the movement behavior components were calculated to describe the overall time-use distribution of 24-hour behaviors. In addition, a variation matrix was used to quantify the dispersion of each component, reflecting within-sample variability.
2. Compositional regression analysis. This study applied an isometric log-ratio (ILR) transformation based on a pivot-coordinate approach to the 24-hour movement behavior data (MVPA, LPA, SB, and sleep). The four-part composition was transformed into three orthonormal ILR coordinates. This method preserves the relative nature of compositional data while integrating the relative relationships between different behaviors into a unified regression framework. Based on the transformed ILR coordinates, compositional regression models were constructed to examine the associations between MVPA, LPA, SB, and sleep as relative time-use components and NSSI scores among adolescents. Within this framework, the effect of each behavior can be interpreted as its relative change in relation to the remaining components of the 24-hour composition. The models were adjusted for gender, age, BMI, parental educational level, family economic status, and place of origin. School was included as a random intercept to account for within-school clustering arising from the cluster sampling design, thereby improving the robustness and reliability of statistical inferences.
3. Isotemporal substitution analysis. Previous studies have indicated that a 15-minute change in activity behavior may be associated with changes in health indicators [20]. Therefore, this study used a 15-minute interval as the basic time-reallocation unit to estimate the relative change in NSSI scores when time from one activity component was reallocated to another. Specifically, isotemporal substitution effects were derived by hypothetically reallocating a fixed duration of time between activity components while holding total daily time constant, followed by re-expression of the modified composition using isometric log-ratio (ILR) coordinates. The sample mean composition was used as the reference composition for all reallocation scenarios. The resulting ILR values were then entered into the fitted compositional regression model to obtain predicted NSSI scores, and substitution effects were calculated as the difference between predicted values under the reallocation scenario and those under the reference composition. These estimates represent model-based predicted differences under hypothetical reallocation scenarios and should not be interpreted as observed behavioral changes. Model diagnostics further indicated that the assumptions of residual normality and homoscedasticity were reasonably satisfied.
4. Dose-response analysis. Given that the mean MVPA in the present study was 40.52 minutes and considering the non-negativity constraint of compositional data, changes ranging from −40 to 40 minutes were examined in 5-minute increments to further explore the dose-response relationship between activity behaviors with significant substitution effects and NSSI. A change in NSSI scores was considered statistically significant if the 95% confidence interval (CI) did not include zero. The statistical significance level for all analyses was set at p < 0.05.
5. Sensitivity analysis. To examine the robustness of the primary findings regarding the distributional characteristics of the NSSI outcome, sensitivity analyses were conducted. The NSSI score was recoded as a binary variable (0 = no NSSI; 1 = any NSSI, and compositional logistic regression models using the isometric log-ratio (ILR) transformation were applied within the isotemporal substitution framework. These analyses were used to assess whether the associations observed in the primary compositional linear regression models were consistent under an alternative specification of the outcome variable.

## Results

### Descriptive statistics

A total of 536 junior high school students were included in the study, with a mean age of 12.38 ± 0.60 years and a mean BMI of 19.83 ± 3.85 kg/m². The sample comprised 270 girls (50.37%) and 266 boys (49.63%); 314 students (58.58%) from high-income families and 222 students (41.42%) from low-income families; 190 students (35.45%) with parents whose educational level was middle school or below, 202 students (37.69%) with parents who completed high school or vocational school, and 144 students (26.86%) with parents who had a college degree or higher; and 426 urban students (79.50%) and 110 rural students (20.50%). The mean score of NSSI was 8.53 ± 3.47.

The distribution of 24-hour movement behaviors showed that the arithmetic means of MVPA, LPA, SB, and sleep were 0.77 h/day (3.20%), 2.76 h/day (11.51%), 12.41 h/day (51.72%), and 8.06 h/day (33.57%), respectively. The compositional geometric means were 0.68 h/day (2.81%), 2.64 h/day (11.00%), 12.52 h/day (52.19%), and 8.16 h/day (34.00%), respectively (see Table 1).

**Table 1:** Descriptive characteristics of the sample

Variables	M(SD)/*N*%
Age(yr.)	12.38(0.60)
BMI(kg/m²)	19.83(3.85)
Gender, n, %	
Boy	266(49.63)
Girl	270(50.37)
Family Economic Status, n, %	
High-income	314 (58.58)
Low-income	222 (41.42)
Parental Education Level, n %	
middle school or below	190 (35.45)
high school or vocational school	202 (37.69)
College or higher	144 (26.86)
Place of origin, n %	
Urban	426 (79.5)
Rural	110 (20.5)
NSSI score (SIBS)	8.53 (3.47)
Arithmetic mean (h/day, %)	
MVPA	0.77 (3.20)
LPA	2.76 (11.51)
SB	12.41 (51.72)
Sleep	8.06 (33.57)
Compositional geometric mean (h/day, %)	
MVPA	0.68 (2.81)
LPA	2.64 (11.00)
SB	12.52 (52.19)
Sleep	8.16 (34.00)

Values are arithmetic mean ± SD unless stated otherwise

*MVPA* Moderate-to-vigorous physical activity, *LPA* Light physical activity, *SB* Sedentary behavior

Table 2 presents the variation matrix of the 24‑hour movement behavior composition. The results showed that the log-ratio variance between SB and sleep was the smallest (0.0303), indicating a high degree of relative co-variability between these two behaviors within the time-use composition. In contrast, the log-ratio variances between MVPA and the other behaviors were relatively larger, suggesting greater relative variability of MVPA within the 24-hour time-use composition.

**Table 2:** Compositional variation Matrix of proportions of time spent in PA, SB, and sleep

	MVPA	LPA	SB	Sleep
MVPA	0.0000	0.1522	0.2466	0.2185
LPA	0.1522	0.0000	0.1344	0.1069
SB	0.2466	0.1344	0.0000	0.0303
Sleep	0.2185	0.1069	0.0303	0.0000

*MVPA* Moderate-to-vigorous physical activity, *LPA* Light physical activity, *SB* Sedentary behavior

These abbreviations apply to all subsequent tables

### Compositional data regression analysis

Table 3 presents the results of the compositional regression analysis, examining all 24-h movement behaviors in relation to NSSI scores. After adjusting for covariates, compositional regression analyses were conducted using isometric log-ratio (ILR)-transformed 24-hour movement behavior components as independent variables, with NSSI score as the dependent variable. The results showed that an increased relative proportion of MVPA, accompanied by a proportional decrease in the other behaviors, was significantly associated with lower NSSI scores (*β* = −0.93, *p* = 0.03). In contrast, an increased relative proportion of SB, with a corresponding decrease in the other behaviors, was significantly associated with higher NSSI scores (*β* = 10.37, *p* = 0.001). No significant association was observed between the relative proportion of LPA and NSSI scores (*β* = 1.01, *p* > 0.05), nor between the relative proportion of sleep and NSSI scores (*β* = −0.14, *p* > 0.05).

**Table 3:** Compositional regression analysis of 24-hour movement behaviors and NSSI scores

	*β*	SE	*t*-value	95% CI	*p*-value	Model *P*	Model *R*^2^
ilr-MVPA/(LPA*SB*Sleep)	−0.93	0.44	−2.11	−1.80, − 0.06	0.03	< 0.001	0.107
ilr-LPA/(MVPA*SB*Sleep)	1.01	0.74	1.35	−0.46, 2.47	0.18		
ilr-SB/(MVPA*LPA*Sleep)	10.37	2.12	4.89	6.20, 14.55	0.001		
ilr-Sleep/(MVPA*LPA*SB)	−0.14	0.85	−0.17	−1.81, 1.53	0.87		

The model was adjusted for covariates, including age, gender, parental educational level, family economic status, place of origin, and BMI. *β* values represent the effect of a change in the given behavior relative to the other behaviors on NSSI scores. ilr-MVPA/(LPA*SB* sleep) represents the association strength between MVPA time taken as the first coordinate and NSSI, relative to sleep, SB, and LPA time

### Gender differences in 24-hour movement behaviors and their associations with non-suicidal self-injury scores

**Fig. 2:**
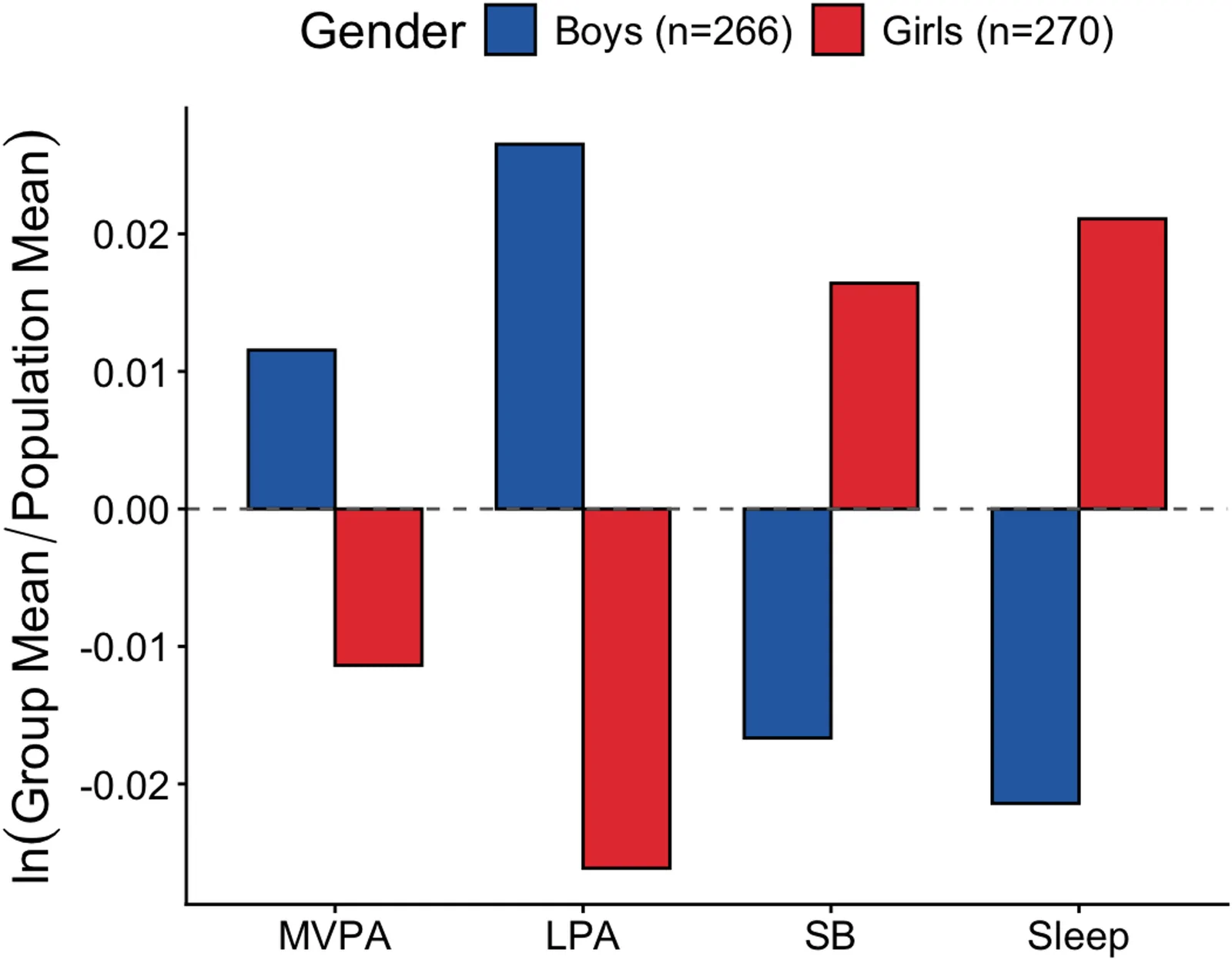
Gender differences in adolescents’ 24-hour movement behaviors

To examine gender differences in 24-hour movement behaviors among adolescents, compositional geometric mean bar charts (Fig. 2) were used to display deviations in the 24-hour movement behavior composition between boys and girls relative to the overall geometric mean, based on log-ratio analysis. The results showed that boys had higher compositional geometric mean values for MVPA and LPA compared with the overall mean, whereas girls exhibited higher compositional geometric mean values for SB and sleep. Among all behavioral components, the largest gender difference was observed in LPA.

**Fig. 3:**
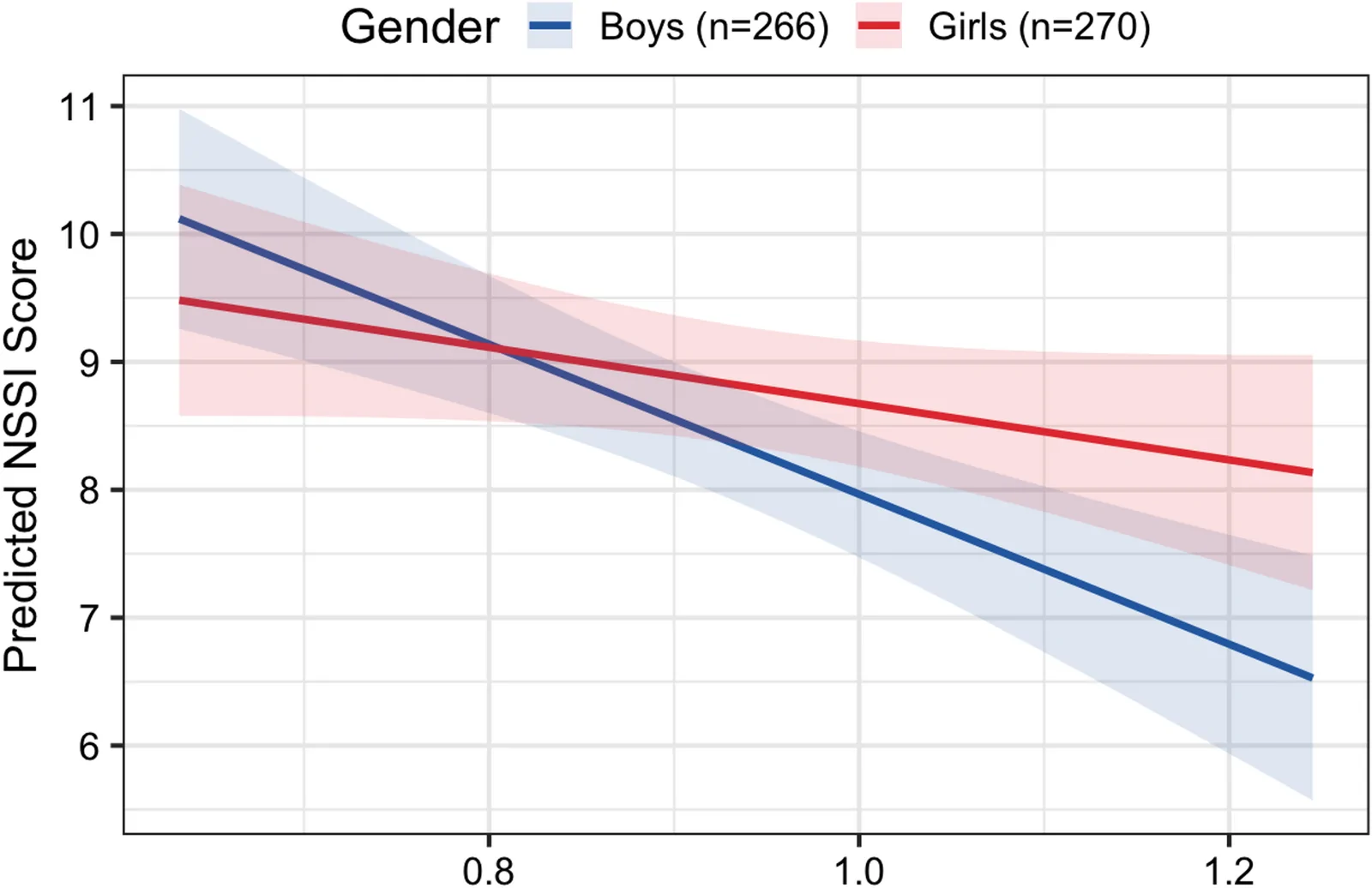
Sleep proportion in 24-hour movement behavior composition

To examine potential sex differences in the associations between 24-hour movement behaviors and NSSI score, formal interaction analyses were conducted to test whether gender moderates these associations. After adjusting for age, BMI, urban residence, parental education level, and household economic status, no significant interactions were observed between MVPA or LPA proportion and gender (all *p* > 0.05). In contrast, a significant interaction was identified between sleep proportion and gender (*β* = 3.66, *p* < 0.05). As shown in Fig. 3, with increasing sleep proportion in the 24-hour movement behavior composition, predicted NSSI scores decreased more markedly among boys, whereas the trend was relatively stable among girls.

### Reallocation of 24-h movement behavior duration and its impact on the prediction of non-suicidal self-injury scores

Table 4 shows the estimated changes in NSSI scores following a 15‑min reallocation between 24‑h movement behaviors. Replacing LPA or SB with MVPA was associated with significant reductions in NSSI scores of 0.31 units (− 0.09 SD) and 0.37 units (− 0.11 SD), respectively. In contrast, replacing MVPA with LPA or SB was associated with significant increases of 0.44 units (0.13 SD) and 0.51 units (0.15 SD), respectively. Replacing sleep with SB was associated with a significant increase in NSSI scores of 0.18 units (0.05 SD), while the reverse substitution was associated with a significant decrease of 0.18 units (− 0.05 SD). Sensitivity analyses using binary logistic regression yielded findings generally consistent with those of the primary compositional regression models, with similar effect directions and no substantial changes in statistical inference. These results further support the robustness of the primary findings.

**Table 4:** Reallocations of 15 min from one movement behavior to another

	MVPA↑	LPA↑	SB↑	Sleep↑
	β (95%CI)	β (95%CI)	β (95%CI)	β (95%CI)
MVPA↓		0.44 (0.05, 0.83) ^*^	0.51 (0.19, 0.83) ^*^	0.33 (− 0.01, 0.66)
LPA↓	−0.31 (− 0.60, − 0.01) ^*^		0.07 (− 0.04, 0.17)	−0.11 (− 0.24, 0.01)
SB↓	−0.37 (− 0.59, − 0.16) ^*^	−0.07 (− 0.16, 0.03)		−0.18 (− 0.25, − 0.11) ^*^
Sleep↓	−0.19 (− 0.42, 0.04)	0.12 (− 0.01, 0.23)	0.18 (0.11, 0.26) ^*^	

The model was adjusted for covariates, including age, gender, parental educational level, family economic status, place of origin, and BMI. ↑ indicates a 15-min increase in the corresponding activity behavior, and ↓ indicates a 15-min decrease. *, *p* < 0.05. Time reallocations were estimated based on the compositional geometric mean of the sample

### Dose-response relationship between 24-h movement behavior reallocations and non-suicidal self-injury scores

**Fig. 4:**
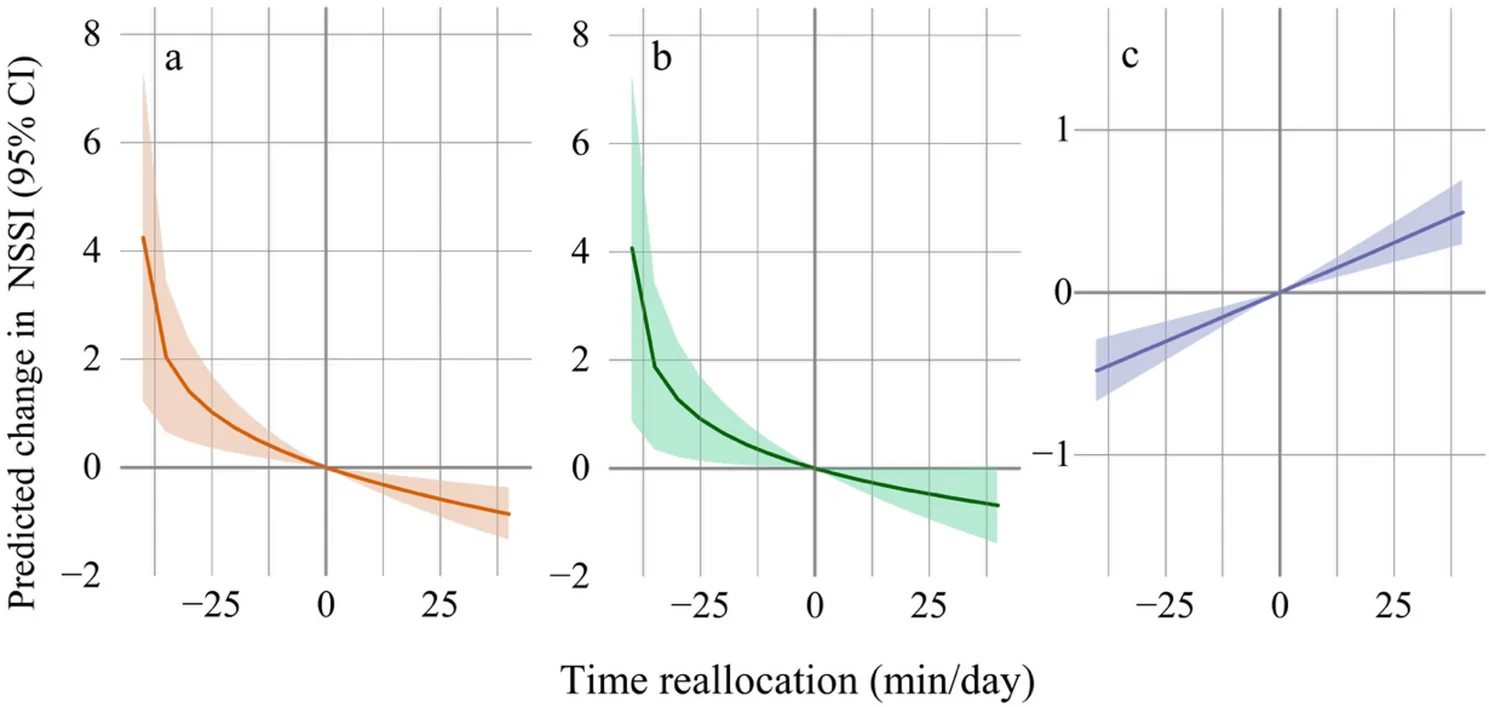
The impact of 24-h isotemporal substitution of movement behaviors on non-suicidal self-injury scores in adolescents. Note: a = isotemporal substitution of MVPA for LPA; b = isotemporal substitution of MVPA for SB; c = isotemporal substitution of SB for sleep

Based on the compositional mean of MVPA in the present study (40.52 min) and the observed patterns in the 15-min substitution results, we used the MVPA mean as the reference point and examined the dose-response relationship between different substitution durations and NSSI scores across a range of − 40 min to 40 min in 5-min increments (see Fig. 4). The results showed that when MVPA replaced LPA or SB, NSSI scores decreased with increasing substitution time across the 5–40 min range. Specifically, reductions ranged from 0.11 (− 0.03 *SD*) to 0.68 (− 0.20 *SD*) units for MVPA replacing LPA, and from 0.14 (− 0.04 *SD*) to 0.86 (− 0.25 *SD*) units for MVPA replacing SB, with the most pronounced decrease observed for MVPA replacing SB.

Conversely, when LPA or SB replaced MVPA, NSSI scores increased with increasing substitution time across the − 5 to − 40 min range. Increases ranged from 0.13 (0.04 *SD*) to 4.07 (1.17 *SD*) units for LPA replacing MVPA, and from 0.15 (0.04 *SD*) to 4.24 (1.22 *SD*) units for SB replacing MVPA. The magnitude of the increase in NSSI scores when LPA or SB replaced MVPA was substantially larger than the magnitude of the decrease when MVPA replaced LPA or SB, indicating an asymmetric dose-response relationship. Substitutions between SB and sleep were largely symmetric. When SB replaced sleep, NSSI scores increased significantly by 0.06 (0.02 *SD*) to 0.49 (0.14 *SD)* units, whereas when sleep replaced SB, NSSI scores decreased significantly by 0.06 (− 0.02 *SD*) to 0.48 (− 0.14 *SD*) units. Furthermore, when MVPA replaced LPA or SB for 20 min, NSSI scores decreased by 0.39 (− 0.11 *SD*) and 0.48 (− 0.14 *SD*) units, respectively. Beyond 20 min (i.e., from 25 to 40 min), the magnitude of the decrease in NSSI scores gradually slowed, with reductions ranging from 0.47 (− 0.14 *SD*) to 0.68 (− 0.20 *SD*) units for MVPA replacing LPA, and from 0.58 (− 0.17 *SD*) to 0.86 (− 0.25 *SD*) units for MVPA replacing SB. These findings indicate that the downward trend in NSSI scores became less steep beyond approximately 20 min of MVPA substitution.

## Discussion

Guided by emotion regulation theory and a 24-hour time-use co-dependency perspective, this study examined the associations of MVPA, LPA, SB, and sleep behaviors with non-suicidal self-injury (NSSI) among adolescents.

The results of the present study showed that Chinese adolescents spent an average of 40.52 min per day in MVPA, 158.35 min in LPA, 751.49 min in SB, and 489.63 min in sleep. In terms of activity distribution, Chinese adolescents showed relatively lower MVPA compared with international studies. This value was comparable to a previous meta-analysis in Chinese adolescents [41] but significantly lower than in Finland (80 min/d) [42] and Germany (51.85 min/d) [43], and only slightly higher than in Brazil (31.30 min/d) [44]. The average daily LPA of 158.35 min was also lower than in Finland, Germany, and Brazil. Average daily SB of 751.49 min was considerably higher than in Sweden (619 min/d) [45] and Finland (480 min/d) [42], while sleep duration was relatively similar across countries. This pattern of low activity and high sedentary behavior aligns with previous findings [46]. However, it should be noted that methodological heterogeneity may exist between this study and the aforementioned studies in terms of accelerometer cut-points, assessment protocols, sample inclusion criteria, and data processing procedures. In addition, within the 24-hour movement behavior composition, MVPA showed greater relative variability compared with other behaviors, whereas SB and sleep exhibited stronger co-variation. In the context of China’s examination-oriented education system, substantial academic workload and fixed instructional schedules may constrain discretionary time, thereby limiting the flexibility of MVPA [47]. Meanwhile, fluctuations in study time may correspondingly displace or compensate for sleep duration [33], resulting in a relatively stronger trade-off between SB and sleep.

From the perspective of emotion regulation theory, 24-hour movement behaviors may influence NSSI through their differential effects on affect regulation capacity, emotional recovery, and stress resilience. Specifically, MVPA may enhance adolescents’ capacity to regulate negative affect by promoting psychological resilience, reducing physiological stress responses, and facilitating adaptive coping strategies. In contrast, excessive sedentary behavior may limit opportunities for emotional recovery and increase exposure to maladaptive cognitive processes such as rumination, thereby increasing vulnerability to emotion dysregulation and self-injurious behaviors. Consistent with this framework, the compositional regression results indicated that reallocating time to MVPA was significantly associated with lower NSSI scores, thereby supporting Hypothesis 1. This finding is consistent with several previous studies examining the relationship between MVPA or PA and NSSI. For example, a longitudinal study by Jung et al. reported that MVPA was associated with reduced NSSI [15]. In addition, a follow-up study indicated that regular PA significantly decreased the risk of NSSI occurrence [48]. A case-based intervention study further demonstrated that PA had a beneficial effect in reducing the frequency of NSSI behaviors [49]. However, these findings are inconsistent with a compositional data analysis study using self-reported questionnaires, which found no significant association between MVPA and adolescent NSSI scores [50]. This discrepancy may be attributable to differences in measurement methods.

In contrast, a higher proportion of SB was significantly associated with increased NSSI scores. This result is consistent with the findings of Guo et al., who reported a positive association between sedentary time and NSSI scores among adolescents [51]. At present, direct evidence on the association between SB and NSSI in adolescents remains limited, with most studies focusing on screen-based leisure SB. For instance, a large-scale study among Chinese adolescents found that excessive screen-based SB and prolonged homework time were significantly associated with an increased risk of NSSI [52]. A compositional data analysis further indicated that higher screen time-based SB within a 24-hour movement behavior framework was associated with increased NSSI risk among adolescents [50]. Notably, evidence also suggests that adolescents with a behavioral pattern characterized by high MVPA combined with low screen time-based SB exhibit the lowest risk of NSSI compared with other behavioral compositions [53]. These findings collectively suggest that maintaining sufficient levels of MVPA and reducing SB may be closely associated with lower NSSI in adolescents.

From a gender-stratified perspective, boys exhibited higher durations of MVPA and LPA than girls, while girls spent slightly more time on SB and sleep. This pattern is consistent with findings from the International Children’s Accelerometry Database (ICAD), which included 27,637 children and adolescents [54]. Such differences may reflect gendered differences in daily routines and activity contexts, with boys more likely to engage in unstructured physical activities and girls exhibiting more structured schedules with relatively higher sedentary exposure [55, 56]. Building on these findings, this study further examined whether sex moderates the association between 24-hour movement behaviors and NSSI. The results of the interaction analysis indicated that the association between sleep proportion and NSSI was significantly moderated by sex, with a stronger negative association observed in boys. Within the framework of emotion regulation theory, adequate sleep is essential for maintaining emotional stability and regulatory functioning, whereas sleep deprivation impairs emotional processing and inhibitory control, both of which are implicated in NSSI [57–59]. Therefore, the stronger protective association observed in boys may indicate potential sex differences in how sleep-related restoration of emotion regulation capacity is associated with NSSI risk. This pattern suggests that sex differences were specific to the sleep–NSSI association rather than a general feature of all movement behaviors. The absence of significant moderating effects for MVPA and sedentary behavior further suggests that their associations with NSSI are relatively stable across gender groups. In contrast, sleep-related regulation processes may be more sensitive to sex differences during adolescence. These findings highlight the importance of considering gender-specific sleep-related mechanisms when designing 24-hour movement behavior–based interventions for adolescent NSSI.

The isotemporal substitution model showed that reallocating 15 min per day to MVPA in place of SB or LPA was significantly associated with lower NSSI scores among adolescents, which verified Hypothesis 2. Comparison across different substitution scenarios indicated that replacing SB with MVPA yielded the largest reduction in NSSI scores, suggesting that reallocating time from SB to MVPA may confer potential benefits in reducing NSSI. This finding is consistent with emotion regulation theory, as reallocating time from sedentary behavior to MVPA may enhance adaptive emotion regulation capacity while simultaneously reducing exposure to behavioral contexts associated with affective dysregulation. In addition, replacing SB with 15 min of sleep was also associated with reduced NSSI scores, although the effect size was smaller. Previous studies have shown that sleep deprivation and insufficient sleep are associated with a higher risk of NSSI among adolescents [60, 61], while extending sleep duration, such as weekend catch-up sleep, has been linked to a lower likelihood of NSSI [62]. In conclusion, based on the findings regarding time reallocation in this study, optimizing physical education class intensity, reducing SB during breaks, and appropriately extending weekend sleep duration can provide feasible ideas and research references for future interventions targeting adolescent NSSI.

Further dose-response analyses revealed that the effects of isotemporal substitution exhibited both symmetric and asymmetric patterns. Specifically, when MVPA replaced other types of behaviors, the reduction in adolescent NSSI scores was relatively gradual. In contrast, when other behaviors substituted MVPA, the increase in NSSI scores was more pronounced. These findings are consistent with previous studies on compositional behavior substitution [20, 63]. Considering the characteristics of the present sample, adolescents had an average daily sedentary time of 751.49 min, whereas the average daily MVPA was only 40.52 min. From the perspective of time composition, a 5-minute reduction in MVPA accounts for more than one-eighth of its total daily duration, whereas the same time increment represents only approximately one one-hundred-and-fiftieth of total SB time. Combined with our results, adolescent NSSI risk is more prone to increase and less responsive to decrease, which highlights the necessity of keeping regular daily MVPA among youngsters. In contrast to the above asymmetric substitution pattern, this study found a symmetric pattern in the isotemporal substitution between sleep and SB in relation to adolescent NSSI. Specifically, whether SB replaced sleep or sleep replaced SB, the magnitude of change in NSSI was largely comparable, differing mainly in direction. This suggests that sleep and SB may exert relatively similar effects on adolescent NSSI outcomes. However, the variability matrix indicated that, in practical substitution scenarios, reallocating time between sleep and SB may be more feasible in real-world settings. Accordingly, reducing SB, such as pre-sleep screen exposure, could help optimize daily schedules by increasing sleep duration, thereby promoting a more balanced 24-hour behavioral composition. Such adjustments may have potential implications for the prevention of adolescent NSSI.

This study found that when MVPA was substituted for LPA or SB, the rate of reduction in NSSI scores gradually slowed after reaching 20 min. This suggests that, based on the current level of MVPA (40.52 min/day), an additional 20 min of MVPA per day (i.e., approximately 60 min/day in total) may be associated with the most favorable effect in reducing NSSI. This pattern may reflect a level at which the emotion-regulatory benefits of MVPA appear to plateau, suggesting that sufficient engagement in MVPA may be required to achieve meaningful reductions in emotional vulnerability associated with NSSI. This finding is consistent with the World Health Organization and national physical activity guidelines, which recommend at least 60 min/day of MVPA for adolescents [64, 65]. From a compositional data analysis perspective, the health benefits of MVPA depend not only on its absolute duration but also on the relative reallocation of time among other movement behaviors [66]. Therefore, encouraging adolescents to replace more than 20 min of SB or LPA with MVPA each day may represent an effective strategy for reducing NSSI.

### Limitations and strengths

This study examined NSSI in adolescents from a novel 24-hour movement behavior perspective, although several limitations should be acknowledged. First, the cross-sectional design precludes any inference of causal relationships. In addition, the use of cluster sampling in a single province may limit the generalizability of the findings. NSSI data were collected retrospectively over the past six months using self-report measures, which may introduce recall bias and do not rule out the possibility of reverse causation between movement behaviors and NSSI. Second, sleep duration was determined by combining sleep logs with accelerometer data. Although this approach improves objectivity and data completeness, it may still involve minor recall errors and uncertainty in identifying non-wear periods. The exclusion of invalid records reduced the final sample size and may have introduced selection bias. Finally, some results showed borderline statistical significance with small effect sizes and limited explanatory power, suggesting that additional psychosocial factors not assessed in the present study may contribute to adolescent NSSI. For example, academic stress [67, 68] and internet addiction [14, 69] may influence NSSI directly or indirectly through emotion regulation difficulties. Future studies should adopt large-scale longitudinal designs and more precise objective monitoring tools to clarify the causal and dynamic relationships between 24-hour movement behaviors, emotion regulation processes, and adolescent NSSI.

## Conclusion

This study found that Chinese adolescents generally exhibit insufficient PA and prolonged SB time. Within the 24-hour movement behavior composition, allocating more time to MVPA, relative to other behaviors, was significantly associated with lower NSSI scores, whereas allocating more time to SB was significantly associated with higher NSSI scores. Reallocating time from SB to MVPA or sleep, as well as from LPA to MVPA, was associated with lower NSSI scores, with the most pronounced effect observed when SB was replaced by MVPA. These findings provide new insights for the development of PA-based interventions targeting adolescent NSSI and offer empirical evidence that optimizing the allocation of 24-hour movement behaviors may contribute to healthier emotion regulation and lower NSSI risk among adolescents.

## Supplementary Information

Supplementary Material 1.

## Data Availability

All data supporting the findings of this study are available from the corresponding author upon reasonable request.
